# Risk factors, and outcomes of patients with carbapenem-resistant *Enterobacterales* bloodstream infection: an eight-year case-case-control study

**DOI:** 10.3389/fcimb.2025.1734801

**Published:** 2026-01-12

**Authors:** Haifang Kong, Yong Liu, Yaqing Wang, Ling Yang, Qianqian Chen, Yanchun Li, Zuoliang Dong, Zhidong Hu, Yamin Chai, Xiuyu Wang, Hua Yan

**Affiliations:** 1 Department of Laboratory Medicine, Tianjin Medical University General Hospital, Tianjin, China; 2 Department of Neurosurgery, Huanhu Hospital Affiliated to Tianjin Medical University, Tianjin, China; 3 Tianjin Key Laboratory of Cerebral Vascular and Neurodegenerative Diseases, Tianjin Neurosurgical Institute, Tianjin Huanhu Hospital,Tianjin, China; 4 Qingdao Eighth People’s Hospital, Shandong, China; 5 Department of Neurosurgery, Tianjin First Central Hospital, School of Medicine, Nankai University, Tianjin, China; 6 The Fourth Central Hospital Affiliated to Tianjin Medical University, Tianjin, China

**Keywords:** bloodstream infection, carbapenem-resistant *Enterobacterales*, case-case-control study, outcomes, risk factor

## Abstract

Carbapenem-resistant *Enterobacterales* bloodstream infection (CRE-BSI) represents a major and urgent challenge to global public health. Some patients with CRE-BSI have a greater risk for poor clinical outcomes, thus identifying risk factors for CRE-BSI is required to determine the most at-risk populations. Here, we investigated risk factors for CRE-BSI by conducting a retrospective case-case-control study at Tianjin Medical University General Hospital, between 2017 and 2024. A total of 144 patients with CRE-BSI were enrolled in this case-case-control study. Each case was matched simultaneously to a patient with carbapenem-susceptible *Enterobacterales* BSI (CSE-BSI) and a control patient with non-*Enterobacterales* bacteremia in a 1:1:1 ratio. This design facilitated the analysis of risk factors and a comparison of 30-day survival outcomes among groups. Multivariable logistic regression identified distinct risk factor profiles for different infections. Surgical history emerged as an independent risk factor for *Enterobacterales*-BSI. Independent risk factors for CRE-BSI encompassed prior exposure to third-generation cephalosporins (OR = 1.94), carbapenems (OR = 3.45), quinolones (OR = 2.54), and glucocorticoids (OR = 2.55), in addition to a history of surgery (OR = 2.44) and gastric tube insertion (OR = 2.45). In-hospital mortality for CRE-BSI reached 52.8%. Furthermore, arterial catheter use (OR = 2.50) was identified as an independent risk factor for in-hospital mortality in patients with CRE-BSI. Cox proportional hazards modeling revealed several independent predicators of 30-day mortality: patient group (HR = 1.37; 95% CI, 1.01–1.86; p = 0.04), age ≥ 65 years (HR = 0.43; 95% CI, 0.20–0.93; p = 0.03), respiratory diseases (HR = 3.17; 95% CI, 1.54–6.51; p = 0.002), and digestive system diseases (HR = 1.79; 95% CI, 1.03–3.10; p = 0.04). Thus, a comprehensive evaluation of underlying diseases, antibiotic usage, and invasive procedures is required to reduce CRE-BSI-associated mortality. Given the notable morbidity and mortality, as well as constrained therapeutic options, associated with CRE-BSI, identifying risk factors for CRE-BSI is urgently required for effective disease prevention and to develop novel therapeutic strategies.

## 1 Introduction

The emergence of antimicrobial resistance (AMR) has become a major public health threat (Chioro et al., 2015), with AMR incidence increasing globally due to inappropriate antibiotic use and poor infection control practices. Carbapenem-resistant *Enterobacterales* (CRE) are an especially concerning AMR threat due to their resistance to several last-resort antibiotics, leading to high mortality (World Health Organization, 2017). CRE infections are now among the most frequent and difficult-to-treat infections due to limited effective antimicrobial agent availability (Chotiprasitsakul et al., 2019, European Centre for Disease Prevention and Control (2018), World Health Organization, 2017). In 2015, it was estimated that over 2,000 patients died from infections caused by CRE, particularly bloodstream infections (BSI), in Europe, with the number of attributable deaths increasing more than six-fold between 2007 and 2015 (Cassini et al., 2019). Accordingly, the Centers for Disease Control and Prevention (CDC) assigned CRE the highest threat level, requiring public attention (Centers for Disease Control and Prevention, 2019).

Increased carbapenem use in hospitals has led to increased CRE prevalence, thus constituting a serious public health threat. A report published by the World Health Organization (WHO) and the European Centre for Disease Prevention and Control (ECDC) revealed that between 2017 and 2021, the prevalence of carbapenem resistance in *Klebsiella pneumoniae* isolates increased from 0% to 20% European Centre for Disease Prevention and Control and World Health Organization (2023). In Brazil, CRE prevalence increased from almost zero between 1995–1999, to >20% between 2015–2019 (Ferreira et al., 2020). In the United States, the prevalence of carbapenem resistance among *Klebsiella pneumoniae*, *Enterobacter* spp., and *Escherichia coli* (*E. coli*) bacteremia were 9.7%, 2.2%, and 0.1%, respectively (Satlin et al., 2017). The SENTRY study further demonstrated a statistically significant increase in CRE rates across Latin America over a 20-year period, increasing from 0.6 to 2.9% (Castanheira et al., 2019). According to the China Antimicrobial Surveillance Network (CHINET), the resistance rates of *Enterobacterales* isolates to imipenem and meropenem increased from 3.1% and 2.1% in 2005 to 11.1% and 10.8%, respectively, in 2024 (CHINET, 2024). BSI is one of the most severe nosocomial infections, with *Enterobacterales* being the most common associated pathogen (Zhou et al., 2024), and patients with CRE-BSI demonstrate significantly higher mortality (de Araujo et al., 2025; Trecarichi and Tumbarello, 2017), making it a critical clinical threat.

CRE develops antibiotic resistance through several mechanisms, with the production of carbapenemases—enzymes that degrade carbapenem antibiotics—being the primary mechanism (Lutgring and Limbago, 2016). Carbapenemases are primarily categorized into three classes. Class A features enzymes like the Klebsiella pneumoniae carbapenemase (KPC); Class B comprises metallo-β-lactamases, including the New Delhi metallo-β-lactamase (NDM) and imipenemase (IMP); and Class D is represented by oxacillinases, such as OXA-48. Antibiotic resistance in CRE is also mediated by two other mechanisms: the overexpression of efflux pumps and mutations in outer membrane proteins. KPC carbapenemase is widely found in the United States and Europe (Albiger et al., 2015; Iovleva and Doi, 2017), NDM carbapenemase is most commonly found in Indian, Romania, Spain, and Hungary (van Duin and Doi, 2017; Yong et al., 2009), and OXA-48-like carbapenemase is found in Turkey and surrounding countries (van Duin and Doi, 2017). In China, KPC-2 was the most prevalent carbapenemase among CRE strains isolated from adult patients (70.3%), and NDM among the CRE strains isolated from children (49.0%) (Han et al., 2020). Carbapenemase-encoding genes are often harbored on mobile genetic elements like plasmids, which facilitates their transfer between bacteria, significantly accelerating the spread of carbapenem resistance. Although novel antibiotics, such as ceftazidime-avibactam, have been used to treat CRE infection, they remain ineffective against NDM-producing CRE (Abe et al., 2022). Therefore, identifying and intervening in the risk factors for CRE-BSI is crucial to reducing its incidence among hospitalized patients and ultimately improving clinical outcomes.

While a number of studies have explored risk factors associated with CRE infection, most have employed a case-control design with patients infected with carbapenem-susceptible *Enterobacterales* (CSE) as the control group. Directly comparing CRE cases to CSE may introduce selection bias, which can lead to the false identification of antibiotics as risk factors or an overestimation of the odds ratios of some antibiotics (Behar et al., 2008; Harris et al., 2002). To mitigate this bias, a case-case-control design was implemented, featuring two control groups: patients with CSE and those infected with non-*Enterobacterales* bacteria. This study sought to define risk factors for CRE-BSI, with the goal of informing clinical strategies for the early identification of high-risk patients and the implementation of timely measures.

## 2 Materials and methods

### 2.1 Study design and patient population

**Figure 1:**
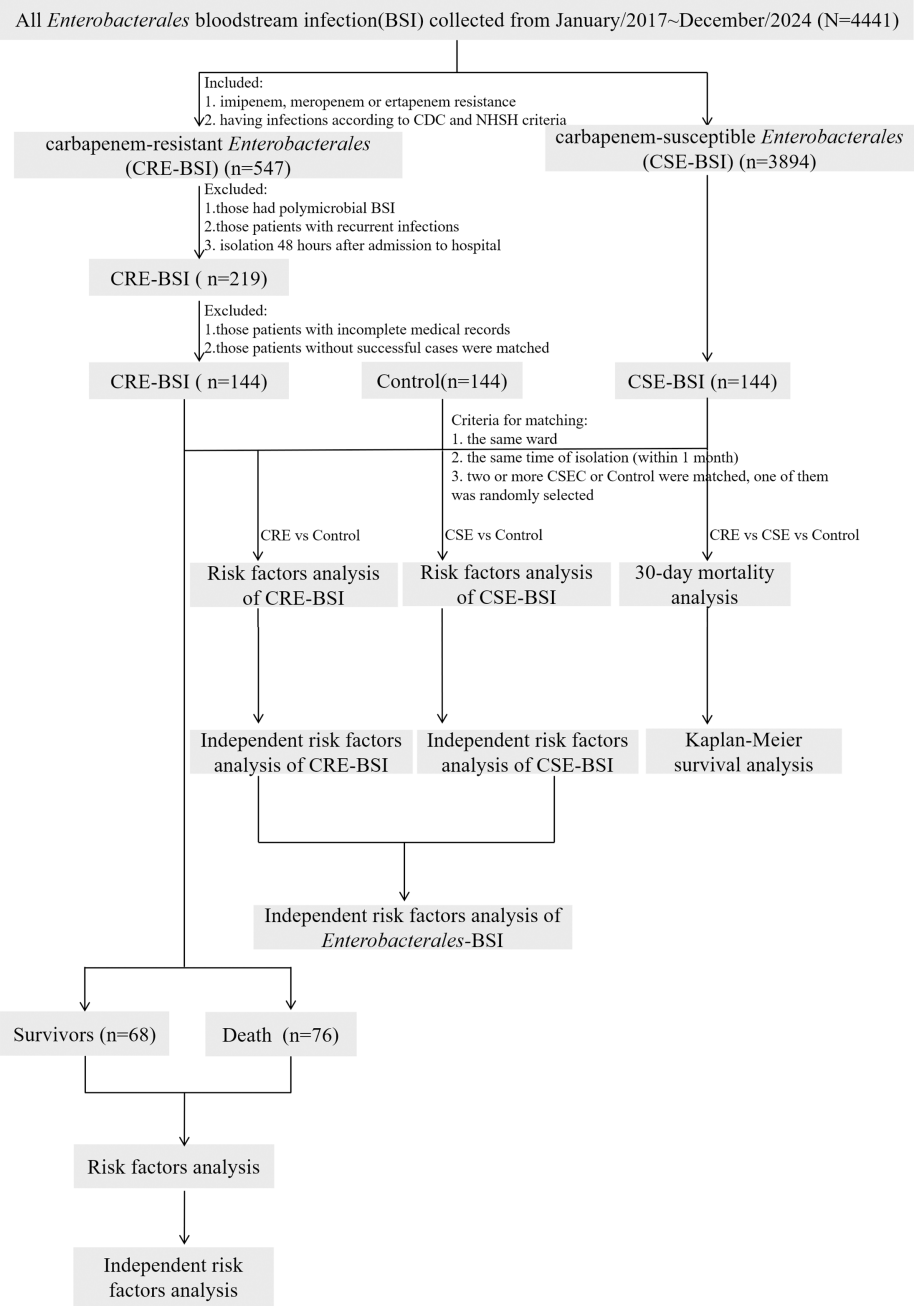
Flowchart of sample selection, comparison and value. From 4441 samples, a total of 144 CRE-BSI isolates were selected for further analysis. A matched CSE-BSI group and control group were used to analyze the risk factors for CRE-BSI and CSE-BSI, then the risk factors of *Enterobacterales*-BSI were obtained. The risk factors for 30-day mortality of CRE-BSI were also analyzed. According to the final outcome of hospitalized patients, the 144 CRE-BSI isolates were divided into survivors and death to analyze the risk factors for in-hospital mortality. CRE, carbapenem-resistant *Enterobacterales*; CSE, carbapenem-susceptible *Enterobacterales*; CDC, Centers for Disease Control and Prevention; NHSN, National Healthcare Safety Network. BSI, bloodstream infection.

We conducted a continuous 8-year, 1:1:1 matched case-case-control study at Tianjin Medical University General Hospital, a 2,468-bed tertiary care center in China, during which a total of 144 clinical CRE-BSI isolates were collected from January 2017 to December 2024 (**Figure 1**). According to the CDC, BSIs are classified by the isolation of pathogenic organisms from blood culture tests (Calandra and Cohen, 2005). Only the first isolate per patient was included. Nosocomial infections for all groups (CRE, CSE, and control) were defined as those identified ≥48 h after admission.

Case groups were defined as hospitalized patients with CRE-BSI or CSE-BSI. Key exclusion criteria included: (1) infection onset before or within 48 hours of admission, (2) polymicrobial BSI, (3) recurrent infection; (4) incomplete medical records, and (5) absence of a matched CSE-BSI.

### 2.2 Case and control selection

This case-case-control study assessed the risk factors and outcomes associated with CRE-BSI (Kaye et al., 2005; Ng et al., 2014; Zhou et al., 2024). This study employed two case-control comparisons, matching CRE-BSI and CSE-BSI inpatients to a common control group.

The CRE group included inpatients with a positive blood culture for *Enterobacterales* resistant to any of the carbapenems (imipenem, meropenem, or ertapenem).

The CSE group consisted of inpatients with bacteremia caused by *Enterobacterales* susceptible to all these carbapenems.

The control group comprised inpatients with confirmed bacteremia from non-*Enterobacterales* pathogens, including Gram-positive bacteria, Gram-negative bacteria such as *Acinetobacter baumannii, Pseudomonas aeruginosa*, and *stenotrophomonas maltophilia*, as well as fungi.

In our case-case-control study, we adopted a 1:1:1 matched design (CRE: CSE: control) based on admission to the same hospital ward and during the same period (within 1 month) as the CRE-BSI group. When multiple eligible controls (CSE-BSI or control groups) were available, one was selected per case using a computer-generated random number sequence, and unmatched isolates were excluded.

### 2.3 Antimicrobial susceptibility testing

We performed blood cultures using the Bactec™ FX 50 system (Becton Dickinson, USA). Isolate identification was carried out by matrix-assisted laser desorption ionization time-of-flight (MALDI-TOF) mass spectrometry (BioMérieux, France), and antimicrobial susceptibility testing was conducted on the Vitek 2-compact system (BioMérieux, France). All susceptibility results were interpreted based on Clinical and Laboratory Standards Institute standards (CLSI-M100) (CLSI, 2023), with *E. coli* ATCC 25922 and *Pseudomonas aeruginosa* ATCC 27853 serving as control strains.

### 2.4 Data collection

Medical records and collected patient information were reviewed as previous reported (Kong et al., 2024; Ng et al., 2014; Stuever et al., 2022). The following clinical information was collected from the medical records of three patient groups: demographics (sex, age, department, previous hospitalization, intensive care unit [ICU] admission, length of hospital stays [LOS], Acute Physiology and Chronic Health Evaluation II [APACHE II] and Sequential Organ Failure Assessment [SOFA] scores), clinical characteristics (comorbidities, microbiological characteristics, mortality), antimicrobial exposure (third–generation cephalosporins, carbapenems, β-lactam inhibitor compounds, aminoglycosides, quinolones, tigecycline, and so on), within 3 months before a positive blood culture. Additional data including surgical history, invasive procedures (mechanical ventilation, various catheters and drainage tubes), within 1 month before a positive blood culture.

All comorbidities and underlying conditions were based on clinically confirmed diagnoses. These included disorders of the respiratory, hepatobiliary, urinary, cardiovascular, digestive, and central nervous systems, as well as diabetes mellitus and malignancies, among others.

Antibiotic exposure was treated as a binary variable (yes/no), defined by any documented administration in the 3 months before bacteremia onset. This included outpatient prescriptions, inpatient regimens initiated after admission but before BSI, and prophylactic courses.

The primary clinical outcomes were assessed by: 30-day mortality (death within 30 days of the first positive blood culture), in-hospital mortality (death occurring during the hospitalization following the first positive blood culture), and LOS (from admission to discharge).

### 2.5 Statistical analysis

All statistical analyses were performed with SPSS 26.0 (IBM Corporation). Continuous data were assessed for normality using the Shapiro-Wilk test. Based on the distribution, Numerical data were presented as mean ± standard deviation (SD) and were compared using Student’s t-test (for two groups); Numerical data were reported as median with interquartile range (IQR) and were compared using the Mann-Whitney U test (for two groups) or the Kruskal-Wallis H test (for three groups). Categorical variables, expressed as numbers and percentages, were compared using the chi-square or Fisher’s exact test, as appropriate. We calculated the p-values, odds ratios (OR), and corresponding 95% confidence intervals (95% CI) for all variables. Variables demonstrating statistical significance (two-tailed p ≤ 0.05) in the univariate analysis were advanced to a multivariable logistic regression model. Survival on 30 days was plotted as Kaplan–Meier curves and compared using the Cox proportional hazards modeling. Assessment of the proportional hazards assumption using Schoenfeld residuals confirmed the validity of the Cox model (**Table 1**, global test p = 0.44). A p-value of ≤ 0.05 was defined as statistically significant, and we assessed the final independent risk factors for multicollinearity. All figures were generated using GraphPad Prism10.0.

**Table 1:** Assessment of the proportional hazards assumption via Schoenfeld residuals.

Variable name	Variable	Chis q	df	p
Group	group	0.128443	1	0.720052
Sex	sex	0.184673	1	0.667387
Age	age	2.1567	1	0.141949
Age65	age65	0.591275	1	0.441926
Respiratory diseases	A1	2.209116	1	0.137197
Hepatobiliary diseases	A2	0.000653	1	0.979608
Urinary system diseases	A3	1.524198	1	0.216985
Circulatory diseases	A4	0.026684	1	0.87024
Digestive system diseases	A6	1.357945	1	0.243894
Diabetes mellitus	A7	0.146324	1	0.702073
Low immunity	A9	0.257604	1	0.611771
3rd-generation Cephalosporin	A11	1.878115	1	0.170549
β-lactam inhibitor	A12	0.049002	1	0.824809
Aminoglycosides	A13	2.544748	1	0.110662
Quinolones	A14	0.548313	1	0.459008
Macrolides	A16	0.084675	1	0.771059
Antifungal drugs	A18	0.450931	1	0.501893
Surgical history	A21	0.513654	1	0.473562
Mechanical ventilation	A22	2.404558	1	0.120982
Arterial catheters	A24	1.992796	1	0.158049
Drainage tube insertion	A26	0.266614	1	0.605612
Gastric tube insertion	A27	1.72867	1	0.188581
	GLOBAL	22.28574	22	0.442948

## 3 Results

### 3.1 Clinical and demographic profiles of patients with CRE-BSI (2017-2024)

Over the eight-year study period, 144 patients with CRE-BSI were identified and subsequently matched to 144 patients with CSE-BSI and 144 controls with non-*Enterobacterales* bacteremia. The majority of CRE-BSI cases (46%; 66/144) were treated in the ICU, while 40% (58/144) and 14% (20/144) were admitted to general medical and surgical wards, respectively.

**Figure 2:**
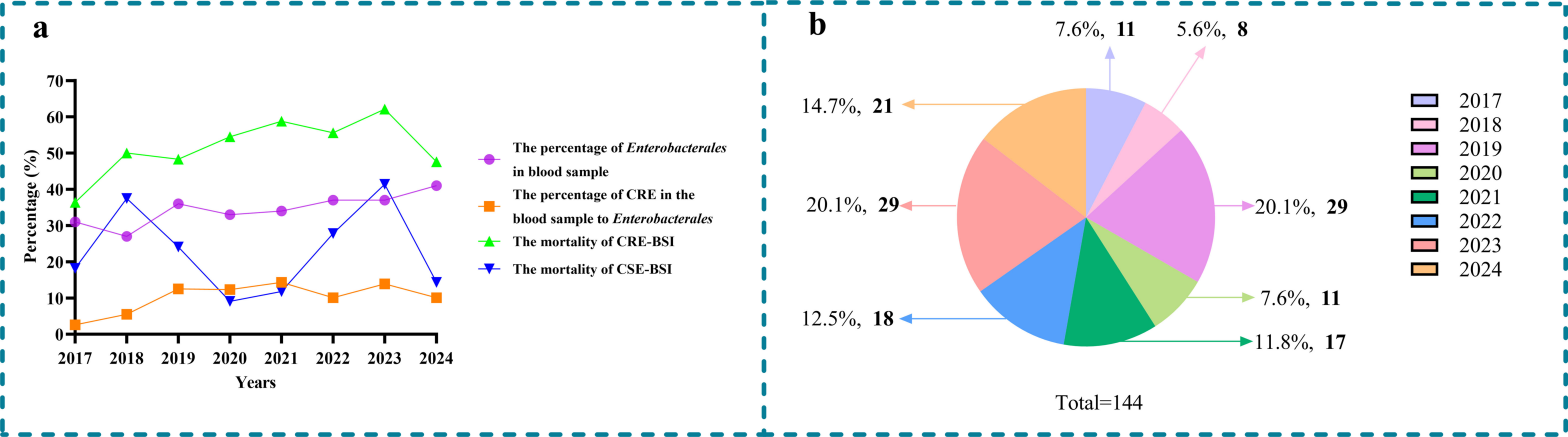
**(a)** Trends in prevalence and mortality of *Enterobacterale* bloodstream infection. **(b)** The proportion and number (black) of CRE-BSI strains isolated over the 8-year study period. CRE, carbapenem-resistant Enterobacterale; CSE, carbapenem-susceptible Enterobacterale; BSI, bloodstream infection.

The percentage of *Enterobacterales* in the positive blood culture fluctuated between 31% and 41% from 2017 to 2024. The percentages of CRE-BSI in *Enterobacterales* bloodstream infection from 2017 to 2024 were 2.6, 5.5, 12.5, 12.3, 14.3, 10.1, 13.9, and 10.1%, respectively, increasing from 2.6% to 14.3% and then dropping to 10.1% (**Figure 2A**). Additionally, the in-hospital mortality was significantly higher in patients with CRE-BSI than in those with CSE-BSI (52.8% vs. 24.3%, p < 0.001). From 2017 to 2024, the in-hospital mortality of CRE-BSI exhibited a fluctuating trend, rising from 36.4% to a peak of 62.1% before declining to 47.6% (**Figure 2A**). As shown in **Figure 2B**, which presents the annual number and proportion of CRE-BSI cases, the majority of strains were isolated in 2019 and 2023.

### 3.2 Antimicrobial susceptibility of CRE-BSI and CSE-BSI

Among the 144 CRE isolates, *Klebsiella pneumoniae* (KP) was the predominant species (81.9%, 118/144), followed by *E. coli* (10.4%, 15/144). The results of the antimicrobial susceptibility testing are summarized in **Table 2**. The CRE isolates exhibited extremely high resistance to most agents. Resistance rates were 100% to cefuroxime, ceftriaxone, and piperacillin/tazobactam; over 89% to ceftazidime, cefepime, amoxicillin/clavulanic acid, cefoperazone/sulbactam, aztreonam, gentamicin, ciprofloxacin, and levofloxacin. Resistance was lower to amikacin (60.4%), tigecycline (23.6%), and colistin (1.4%). Compared to CSE isolates, CRE isolates demonstrated significantly higher resistance rates across most antimicrobial classes (p < 0.001).

**Table 2:** The antibiotic resistance of CRE-BSI and CSE-BSI groups.

Antibiotics	CRE-BSI(n=144)	CSE-BSI(n=144)	χ^2^	*p*
Amoxicillin/clavulanic acid	142/144(98.6%)	13/144(9.0%)	232.5	<0.001
Cefoperazone/sulbactam	138/144(95.8%)	5/144(3.5%)	245.7	<0.001
Piperacillin/tazobactam	144/144(100%)	6/144(4.2%)	265.0	<0.001
Cefuroxime	144/144(100%)	63/144(43.8%)	112.7	<0.001
Ceftazidime	141/144(97.9%)	24/144(16.7%)	194.3	<0.001
Ceftriaxone	144/144(100%)	60/144(41.7%)	118.6	<0.001
Cefepime	141/144(97.9%)	34/144(23.6%)	166.7	<0.001
Aztreonam	135/144(93.8%)	34/144(23.6%)	146.1	<0.001
Amikacin	87/144(60.4%)	2/144(1.4%)	117.5	<0.001
Gentamicin	129/144(89.6%)	46/144(31.9%)	100.3	0.001
Tobramycin	95/144(65.9%)	14/144(9.7%)	96.8	<0.001
Ciprofloxacin	130/144(90.3%)	77/144(53.5%)	48.2	<0.001
Levofloxacin	132/144(91.7%)	58/144(40.3%)	84.7	<0.001
Trimethoprim/ sulfamethoxazole	81/144(56.3%)	62/144(43.1%)	5.01	0.03
Colistin	2/144(1.4)	0/144(0)	2.01	0.16
Tigecycline	34/144(23.6)	5/144(3.5)	24.9	<0.001

CRE, carbapenem-resistant *Enterobacterales*; CSE, carbapenem-susceptible *Enterobacterales*; BSI, bloodstream infection. Statistical test: the chi-square test.

### 3.3 Comparison of the CRE-BSI group to the control group

The baseline demographic and clinical characteristics of the three patient groups are summarized in **Table 3**. The groups were similar in terms of gender, age, APACHE II, and SOFA scores. However, significant differences were observed in prior hospital stays (H = 44.16, p < 0.001), ICU stays (H = 22.53, p < 0.001), and LOS (H = 12.52, p = 0.002).

**Table 3:** Demographic characteristics for 144 patients with CRE-BSI, 144 patients with CSE-BSI and 144 control patients.

Variable	CRE-BSI (N = 144) (n, %)	CSE-BSI (N = 144) (n, %)	Control (N = 144) (n, %)	χ^2^/H	*p*
Sex, male	89(61.8)	87(60.4)	74(51.4)	3.78	0.15
Age (years), median (IQR)	67.5(55.5, 80)	68.5(55, 79)	70(57, 81)	1.56	0.46
Age>65	81(56.3)	84(58.3)	90(62.5)	1.21	0.55
APACHII	22(14, 25)	12(2.5, 23.5)	18(2.94, 23)	5.17	0.08
SOFA	9(5.5, 11.25)	9(5, 14)	9(5.5, 11.5)	0.14	0.93
Prior hospital stays median (IQR)	22(12, 42.5)	10.5(4, 22)	12(4, 25.75)	44.16	<0.001
ICU stays, median (IQR)	9.5(0, 31)	0(0, 7)	0(0, 12)	22.53	<0.001
LOS (days), median (IQR)	41(20.25, 70)	28(16.25, 48.75)	32(14, 62.5)	12.52	0.002

CRE, carbapenem-resistant *Enterobacterales*; CSE, carbapenem-susceptible *Enterobacterales*; BSI, bloodstream infection; IQR, interquartile range; APACHII, Acute Physiology and Chronic Health Evaluation II; SOFA, Sequential Organ Failure Assessment; LOS, length of hospital stays. Statistical test: the chi-square test or Kruskal-Wallis H test.

Univariate analysis identified several significant risk factors for CRE-BSI compared to control patients (**Table 4**). These included respiratory diseases (OR = 1.96; 95%CI, 1.17-3.28; p = 0.01); liver diseases (OR = 1.91; 95%CI, 1.20-3.06; p = 0.007); exposure within 3 months prior to bacteremia to specific antibiotics, including third-generation cephalosporins (OR = 2.13; 95%CI, 1.29-3.50; p = 0.003), carbapenems (OR = 4.82; 95%CI, 2.88-8.08; p < 0.001), β-lactam inhibitor compounds (OR = 2.04; 95%CI, 1.27-3.27; p = 0.003), quinolones (OR = 1.96; 95%CI, 1.15-3.33; p = 0.01), glycopeptides (OR = 7.65; 95%CI, 1.71-34.29; p = 0.002), tigecycline (OR = 3.40; 95%CI, 1.90-6.09; p < 0.001), and glucocorticoids (OR = 3.45; 95%CI, 2.05-5.82; p < 0.001); a history of surgery (OR = 2.26; 95%CI, 1.40-3.66; p = 0.001), and invasive procedures within 1 month prior to bacteremia, including mechanical ventilation (OR = 2.75; 95%CI, 1.70-4.45; p < 0.001), central venous catheter insertion (OR = 2.57; 95%CI, 1.57-4.21; p < 0.001), drainage tube insertion (OR = 2.25; 95%CI, 1.25-4.06; p = 0.006), urinary catheter insertion (OR = 2.23; 95%CI, 1.37-3.63; p = 0.001), and gastric tube insertion (OR = 2.48; 95%CI, 1.54-3.99; p < 0.001). Additionally, longer prior hospital stays (p < 0.001), ICU stays (p = 0.001), and LOS (p = 0.02) were significantly associated with CRE-BSI.

**Table 4:** Univariate analyses regarding the risk factors for CRE-BSI compared to control patients.

Variable	CRE-BSI (N = 144) (n, %)	Control (N = 144) (n, %)	χ^2^/U	OR	95%CI *P*	
Underlying disorder						
Respiratory diseases	111(77.1)	91(63.2)	6.63	1.96	(1.17, 3.28)	0.01
Hepatobiliary diseases	89(61.8)	66(45.8)	7.39	1.91	(1.20, 3.06)	0.007
Urinary system diseases	73(50.7)	62(43.1)	1.69	1.36	(0.86, 2.16)	0.19
Circulatory diseases	100(69.4)	97(67.4)	0.15	1.10	(0.67, 1.81)	0.70
Central nervous diseases	51(35.4)	48(33.3)	0.14	1.10	(0.67, 1.78)	0.71
Digestive system diseases	39(27.1)	33(22.9)	0.67	1.25	(0.73, 2.13)	0.41
Diabetes mellitus	41(28.5)	39(27.1)	0.07	1.07	(0.64, 1.80)	0.79
Malignancy	39(27.1)	28(19.4)	2.35	1.54	(0.89, 2.67)	0.13
Antibiotics exposure within 3 months prior to CRE-BSI						
third-generation Cephalosporin	61(42.4)	37(25.7)	8.91	2.13	(1.29, 3.50)	0.003
β-lactam inhibitor	93(64.6)	68(47.2)	8.80	2.04	(1.27, 3.27)	0.003
Carbapenem	82(56.9)	31(21.5)	37.88	4.82	(2.88, 8.08)	<0.001
Aminoglycosides	10(6.9)	9(6.3)	0.06	1.12	(0.44, 2.84)	0.81
Quinolones	49(34.0)	30(20.8)	6.30	1.96	(1.15, 3.33)	0.01
Tigecycline	51(35.4)	20(13.9)	17.96	3.40	(1.90, 6.09)	<0.001
Glycopeptides	14(9.7)	2(1.4)	9.53	7.65	(1.71, 34.29)	0.002
Glucocorticoid	67(46.5)	29(20.1)	22.56	3.45	(2.05, 5.82)	<0.001
Surgical history and invasive procedures within 1 month prior to CRE-BSI						
Surgical history	73(50.7)	45(31.3)	11.26	2.26	(1.40, 3.66)	0.001
Mechanical ventilation	80(55.6)	45(31.3)	17.32	2.75	(1.70, 4.45)	<0.001
Central venous catheter insertion	106(73.6)	75(52.1)	14.29	2.57	(1.57, 4.21)	<0.001
Arterial catheters	70(48.6)	56(39.2)	2.60	1.47	(0.92, 2.35)	0.11
Urinary catheter insertion	102(71.3)	76(52.8)	10.48	2.23	(1.37, 3.63)	0.001
Drainage tube insertion	40(27.8)	21(14.6)	7.51	2.25	(1.25, 4.06)	0.006
Gastric tube insertion	93(64.6)	61(42.4)	14.29	2.48	(1.54, 3.99)	<0.001
Related to hospitalization						
Prior hospital stays median (IQR)	22(12, 42.5)	12(4, 25.75)	6872	—	—	<0.001
ICU stay, median (IQR)	9.5(0, 31)	0(0, 12)	8117	—	—	0.001
LOS (days), median (IQR)	41(20.25, 70)	32(14, 62.5)	8768	—	—	0.02
Death	76(52.8)	33(22.9)	27.29	3.76	(2.26, 6.25)	<0.001

CRE, carbapenem-resistant *Enterobacterales*; ICU, intensive care unit; IQR, interquartile range; LOS, length of hospital stays; —, no result. Statistical test: the chi-square test or Mann-Whitney U test.

Multivariate analysis identified the following independent risk factors for CRE-BSI (**Table 5**): exposure within 3 months prior to bacteremia to third-generation cephalosporins (OR = 1.94; 95%CI, 1.09-3.46; p = 0.03), carbapenems (OR = 3.45; 95%CI, 1.94-6.11; p < 0.001), quinolones (OR = 2.54; 95%CI, 1.36-4.75; p = 0.004), and glucocorticoids (OR = 2.55; 95%CI, 1.42-4.59; p = 0.002); a history of surgery (OR = 2.44; 95%CI, 1.38-4.33; p = 0.002), and gastric tube insertion within 1 month prior to bacteremia (OR = 2.45; 95% CI, 1.40–4.29; p = 0.002).

**Table 5:** Multivariate analyses regarding the independent risk factors for CRE-BSI and CSE-BSI compared to control group.

Variable	β	SE	Wald (χ^2^)	*OR*	95%*CI*	*p*
CRE-BSI cases vs control group						
Carbapenem	1.24	0.29	17.93	3.45	1.94~6.11	<0.001
3rd-generation Cephalosporin	0.66	0.30	5.02	1.94	1.09~3.46	0.03
Quinolones	0.93	0.32	8.52	2.54	1.36~4.75	0.004
Glucocorticoids	0.94	0.30	9.81	2.55	1.42~4.59	0.002
Surgical history	0.89	0.29	9.36	2.44	1.38~4.33	0.002
Gastric tube insertion	0.90	0.29	9.91	2.54	1.40~4.29	0.002
CSE-BSI cases vs control group						
Malignancy	0.65	0.28	5.33	1.90	1.10~3.29	0.02
Surgical history	0.57	0.25	5.28	1.77	1.09~2.89	0.02
Death vs survivors in CRE-BSI group						
Arterial catheters	0.92	0.34	7.11	2.50	1.27~4.89	0.008

CRE, carbapenem-resistant *Enterobacterales*; CSE, carbapenem-susceptible *Enterobacterales;* BSI, bloodstream infection. Statistical test: multivariable logistic regression analysis.

### 3.4 Comparison of the CSE-BSI group to the control group

Univariate analysis of patients with CSE-BSI versus control patients (**Table 6**) identified malignancies and surgical history (within 1 month before bacteremia) as significant factors. These were subsequently confirmed as independent risk factors in the multivariate model: malignancies (OR = 1.90; 95%CI, 1.10-3.29; p = 0.02) and surgical history (OR = 1.77; 95%CI, 1.09-2.89; p = 0.02) (**Table 5**).

**Table 6:** Univariate analyses regarding the risk factors for CSE-BSI compared to control group.

Variable	CSE-BSI (N = 105) (n, %)	Control (N = 105) (n, %)	χ^2^/U	OR	95%CI *P*	
Underlying disorder						
Respiratory diseases	82(56.9)	91(63.2)	1.17	0.77	(0.48, 1.24)	0.28
Hepatobiliary diseases	78(54.2)	66(45.8)	2.00	1.40	(0.88, 2.22)	0.16
Urinary system diseases	64(44.4)	62(43.1)	0.06	1.06	(0.66, 1.69)	0.81
Circulatory diseases	88(61.1)	97(67.4)	1.22	0.76	(0.47, 1.24)	0.27
Central nervous diseases	51(35.4)	48(33.3)	0.14	1.10	(0.67, 1.78)	0.71
Digestive system diseases	21(14.6)	33(22.9)	3.28	0.57	(0.31, 1.05)	0.07
Diabetes mellitus	43(29.9)	39(27.1)	0.27	1.15	(0.69, 1.91)	0.60
Malignancy	48(33.3)	28 (19.4)	7.15	2.07	(1.21, 3.55)	0.007
Antibiotics exposure within 3 months prior to CSE-BSI						
third-generation Cephalosporin	30(20.8)	37(25.7)	0.95	0.76	(0.44, 1.32)	0.33
β-lactam inhibitor	62(43.1)	68(47.2)	0.51	0.85	(0.53, 1.35)	0.48
Carbapenem	24(16.7)	31(21.5)	1.10	0.73	(0.40 ,1.32)	0.29
Aminoglycosides	6(4.2)	9(6.3)	0.63	0.65	(0.23, 1.88)	0.43
Quinolones	29(20.1)	30(20.8)	0.02	0.96	(0.54, 1.70)	0.88
Tigecycline	19(13.2)	20(13.9)	0.03	0.94	(0.48, 1.85)	0.86
Glycopeptides	7(4.9)	2(1.4)	2.87	3.63	(0.74, 17.77)	0.09
Glucocorticoid	27(18.8)	29(20.1)	0.09	0.92	(0.51, 1.64)	0.77
Surgical history and invasive procedures within 1 month prior to CSE-BSI						
Surgical history	67(46.5)	45(31.3)	7.07	1.91	(1.18, 3.10)	0.008
Mechanical ventilation	50(34.7)	45(31.3)	0.39	1.17	(0.72, 1.91)	0.53
Central venous catheter insertion	80(55.6)	75(52.1)	0.35	1.15	(0.72, 1.83)	0.56
Arterial catheters	54(37.5)	56(39.2)	0.08	0.93	(0.58, 1.50)	0.77
Urinary catheter insertion	81(56.3)	76(52.8)	0.35	1.15	(0.72, 1.83)	0.55
Drainage tube insertion	30(20.8)	21(14.6)	1.93	1.54	(0.84, 2.85)	0.17
Gastric tube insertion	64(44.4)	61(42.4)	0.13	1.09	(0.68, 1.74)	0.72
Related to hospitalization						
Prior hospital stays median (IQR)	10.5(4, 22)	12(4, 25.5)	9629	—	—	0.29
ICU stay, median (IQR)	0(0, 7)	0(0, 12)	9723	—	—	0.31
LOS (days), median (IQR)	28(16.25, 48.75)	32(14, 62.5)	9684	—	—	0.33
Death	35(24.3)	33(22.9)	0.08	1.08	(0.63, 1.86)	0.78

CSE, carbapenem-susceptible *Enterobacterales*; BSI, bloodstream infection; ICU, intensive care unit; LOS, length of hospital stays; IQR, interquartile range; —, no result. Statistical test: the chi-square test or Mann-Whitney U test.

### 3.5 Prognostic analysis of CRE-BSI

**Figure 3:**
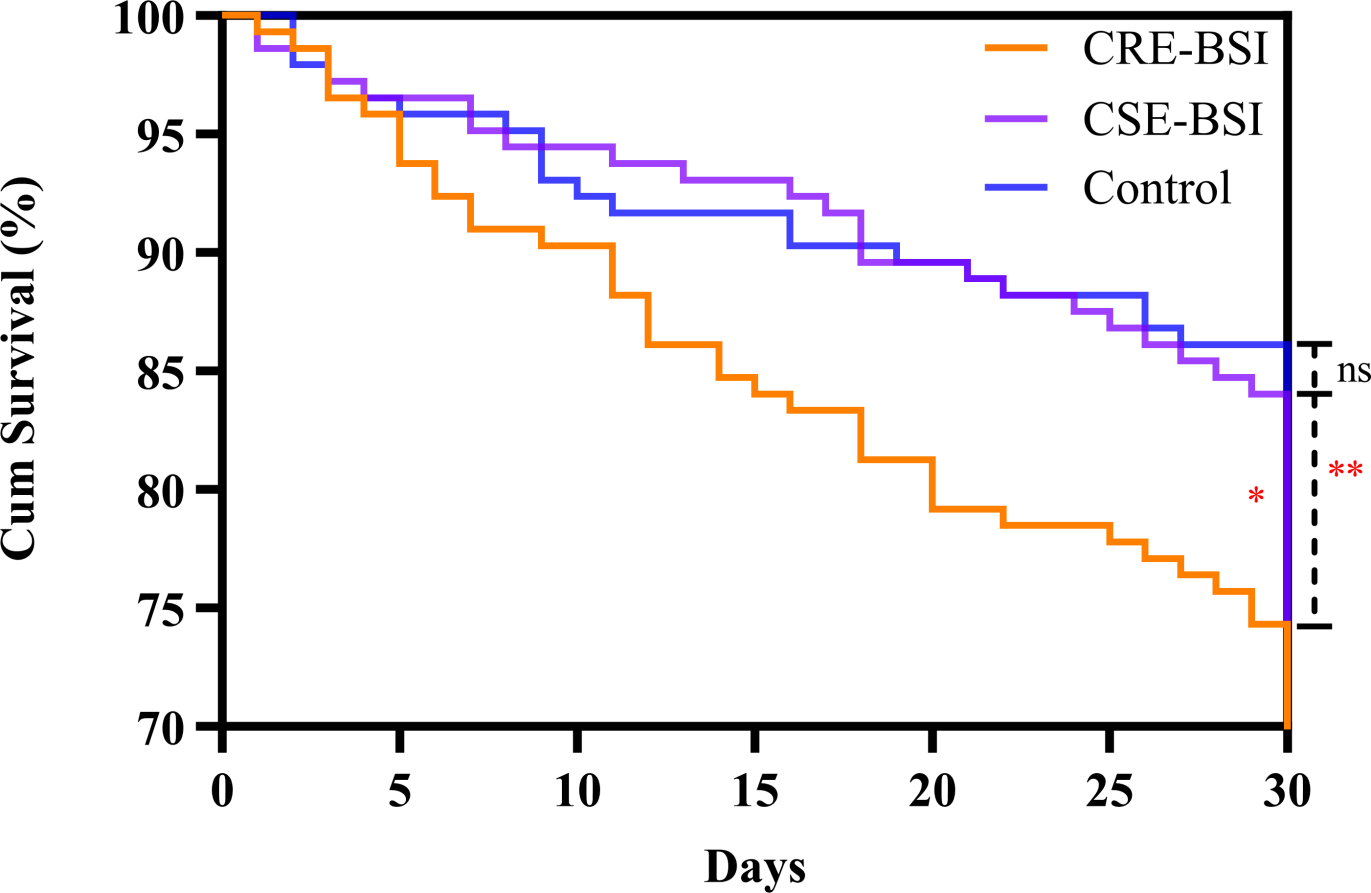
Kaplan–meier curves showing 30-day mortality in the three groups (p=0.02). There was significant difference between the CRE-BSI group and the CSE-BSI group (p=0.04), and there was a significant difference between the CRE-BSI group and the control group (p=0.005), but there was no significant difference between the CSE-BSI group and the control group (p=0.4). CRE, carbapenem-resistant *Enterobacterales*; CSE, carbapenem-susceptible *Enterobacterales*; BSI, bloodstream infection; ns: no significant difference; * represents p < 0.05; ** represents p < 0.01.

Prognostic outcomes differed significantly among the three groups. The 30-day mortality was highest in the CRE-BSI group (25.7%), compared to the CSE-BSI (16.0%) and control (12.5%) groups (p = 0.01). This trend was more pronounced for in-hospital mortality, which was 52.8% of patients in the CRE-BSI group, significantly higher than in the CSE-BSI (28.6%) and control (24.8%) groups (p < 0.001). The three groups also differed significantly in LOS (p = 0.002). Detailed prognostic data are shown in **Table 7**, and Kaplan-Meier survival curves are presented in **Figure 3**.

**Table 7:** Clinical outcomes comparison between the CRE-BSI group, the CSE-BSI group and the control group.

Outcomes	CRE-BSI [n (%)]	CSE-BSI [n (%)]	Control [n (%)]	*p*
30-day mortality	37(25.7)	23(16.0)	18(12.5)	0.02
In-hospital mortality	76(52.8)	35(24.3)	33(22.9)	<0.001
LOS (days), median (IQR)	41(20.25, 70)	28(16.25, 48.75)	32(14, 62.5)	0.002

CRE, carbapenem-resistant *Enterobacterales*; CSE, carbapenem-susceptible *Enterobacterales*; BSI, bloodstream infection; LOS, length of hospital stays; IQR, interquartile range. Statistical test: the chi-square test or Kruskal-Wallis H test.

Cox proportional hazards modeling analysis identified the following independent risk factors for 30-day mortality: patient group (HR = 1.37; 95% CI, 1.01–1.86; p = 0.04), age ≥ 65 years (HR = 0.43; 95% CI, 0.20–0.93; p = 0.03), respiratory diseases (HR = 3.17; 95% CI, 1.54–6.51; p = 0.002), and digestive system diseases (HR = 1.79; 95% CI, 1.03–3.10; p = 0.04). Furthermore, analysis of the CRE-BSI group revealed that in-hospital mortality was associated with arterial catheter use (OR = 2.50; 95%CI, 1.27-4.89; p = 0.007), ICU stays (p = 0.008), and LOS (p = 0.04) in univariate analysis (**Table 8**). Multivariate analysis confirmed arterial catheter use (OR = 2.50; 95%CI, 1.27-4.89; p = 0.008) within 1 month before bacteremia as an independent risk factor for in-hospital mortality in patients with CRE-BSI (**Table 5**).

**Table 8:** Univariable analyses of in-hospital mortality in the CRE group.

Variable	CRE-BSI death (N = 76) (n, %)	CRE-BSI survivors (N = 68) (n, %)	χ^2^/ t/U	OR	95%CI p	
Demographic characteristics						
Sex, male	46(60.5)	43(63.2)	0.11	0.89	(0.45, 1.75)	0.74
Age(years), mean ± SD	65.7 ± 19.6	64.6 ± 16.7	0.34	—	—	0.74
Age>65	41(53.9)	40(58.8)	0.35	0.82	(0.42, 1.59)	0.56
APACHII, mean ± SD	12 ± 23.8	3 ± 19	0.82	—	—	0.43
SOFA, mean ± SD	11 ± 9	3 ± 9.3	0.10	—	—	0.92
Underlying disorder						
Respiratory diseases	63(82.9)	48(70.6)	3.08	2.02	(0.91, 4.46)	0.08
Hepatobiliary diseases	49(64.5)	40(58.8)	0.49	1.27	(0.65, 2.49)	0.49
Urinary system diseases	42(55.3)	31(45.6)	1.34	1.47	(0.76, 2.85)	0.25
Circulatory diseases	53(69.7)	47(69.1)	0.006	1.03	(0.51, 2.09)	0.94
Central nervous diseases	31(40.8)	20(29.4)	2.03	1.65	(0.83, 3.31)	0.15
Digestive system diseases	21(27.6)	18(26.5)	0.02	1.06	(0.51, 2.22)	0.88
Diabetes mellitus	24(31.6)	17(25.0)	0.76	1.39	(0.67, 2.88)	0.38
Malignancy	19(25.0)	20(29.4)	0.35	0.80	(0.38, 1.67)	0.55
Antibiotics exposure within 3 months prior to CRE-BSI						
third-generation Cephalosporin	33(43.4)	28(41.2)	0.07	1.10	(0.57, 2.13)	0.79
β-lactam inhibitor	54(71.1)	39(57.4)	2.95	1.83	(0.92, 3.64)	0.09
Carbapenem	46(60.5)	36(52.9)	0.84	1.36	(0.70, 2.64)	0.36
Quinolones	29(38.2)	20(29.4)	1.22	1.48	(0.74, 2.97)	0.27
Tigecycline	32(42.1)	19(27.9)	3.15	1.88	(0.93, 3.77)	0.08
Glycopeptides	9(11.8)	5(7.4)	0.82	1.69	(0.54, 5.32)	0.36
Glucocorticoid	39(51.3)	28(41.2)	1.48	1.51	(0.78, 2.91)	0.22
Surgical history and invasive procedures within 1 month prior to CRE-BSI						
Surgical history	35(46.1)	38(55.9)	1.39	0.67	(0.35, 1.30)	0.24
Mechanical ventilation	46(60.5)	34(50.0)	1.61	1.53	(0.79, 2.97)	0.20
Central venous catheter insertion	58(76.3)	48(70.6)	0.61	1.34	(0.64, 2.82)	0.44
Arterial catheters	45(59.2)	25(36.8)	7.24	2.50	(1.27, 4.89)	0.007
Urinary catheter insertion	57(75.0)	45(67.2)	1.07	1.47	(0.71, 3.04)	0.30
Drainage tube insertion	23(30.3)	17(25.0)	0.50	1.30	(0.62, 2.72)	0.48
Gastric tube insertion	51(67.1)	42(61.8)	0.45	1.26	(0.64, 2.50)	0.50
Related to hospitalization						
Prior hospital stays median (IQR)	23(12.5, 49)	21.5(12, 34)	2360	—	—	0.37
ICU stays, median(IQR)	14(0, 33.5)	0(0, 26.5)	1938	—	—	0.008
LOS(days),median(IQR)	33(18.25, 60.75)	49.5(28, 84)	2079	—	—	0.04
Antimicrobial Therapy						
Untreated	5(6.6)	7(10.3)	0.65	0.61	(0.19, 2.03)	0.42
Monotherapy with Tigecycline	7(9.2)	8(11.8)	0.25	0.76	(0.26, 2.22)	0.62
Monotherapy with Colistin	0(0)	1(1.5)	1.13	—	—	0.29
Monotherapy without Tigecycline or Colistin	28(36.8)	22(32.4)	0.32	1.22	(0.61, 2.43)	0.57
Combine therapy without Tigecycline or Colistin	5(6.6)	4(5.9)	0.03	1.13	(0.29, 4.38)	0.86
Combine therapy with Tigecycline	10(13.2)	11(16.2)	0.26	0.79	(0.31, 1.98)	0.61
Combine therapy with Colistin	5(6.6)	3(4.4)	0.32	1.53	(0.35, 6.64)	0.57
Combine therapy with Tigecycline and Colistin	4(5.3)	5(7.4)	0.27	0.70	(0.18, 2.72)	0.61
Ceftazidime/avibactam	12(15.8)	7(10.3)	0.95	1.63	(0.60, 4.42)	0.33

SD, standard deviation; CRE, carbapenem-resistant *Enterobacterales*; BSI, bloodstream infection; APACHII, Acute Physiology and Chronic Health Evaluation II; SOFA, Sequential Organ Failure Assessment; LOS, length of hospital stays; IQR, interquartile range; —, no result. Statistical test: Student's t-test, Mann-Whitney U test or the chi-square test.

## 4 Discussion

Patients infected with CRE-BSI demonstrate increased morbidity and mortality, no effective antimicrobial treatment exists for these infections. Thus, identifying risk factors for CRE-BSI etiology and prognosis is essential for reducing incidence and informing optimal therapeutic strategies. The selection of an appropriate control group is critical for accurate assessment of risk factors for CRE-BSI. Using patients with CSE-BSI as controls, a practice common in previous studies, is methodologically problematic. This design inherently assumes a “replacement scenario” where patients are infected exclusively with either CRE or CSE, thereby failing to account for patients infected with other pathogens or those without infection. Patients with CSE constitute a minor fraction of all hospitalized cases and are thus not representative (Harris et al., 2001; Huang et al., 2023; Kaier and Frank, 2013). Furthermore, certain antibiotics may suppress or kill some CSE strains without affecting CRE strains. Consequently, the frequency of antibiotic use in CSE may be reduced, indirectly amplifying the risk factors for CRE (Ng et al., 2014). In addition, few studies employed patients without CRE infection as the control group, without distinguishing between susceptible and resistant groups, making it difficult to independently analyze the risk factors for *Enterobacterales* infection. This study employed a case-case-control design to comprehensively compare patient characteristics among those with CRE-BSI, CSE-BSI, and control group. It confirmed established risk factors while uniquely distinguishing those specific to carbapenem resistance from those common to *Enterobacterales* infection in general, thereby providing potential therapeutic options for CRE-BSI.

We found that Carbapenem-resistant *Klebsiella pneumoniae* was the predominant strain among cases with clinical BSI due to CRE (81.9%), which is slightly higher than that reported in other studies (Chen et al., 2022; Yan et al., 2020). CRE-BSI cases were most frequently identified in the ICU, with the hematology department being the second most common location. This distribution is attributed to the confined environment of the ICU, enhancing the risk of CRE transmission via airborne and contact transmission. Patients in the ICU often have severe comorbidities and require invasive procedures as well as treatment with broad-spectrum antibiotics, factors that contribute to an increased risk of CRE-BSI. Notably, patients with hematologic diseases have a unique vulnerability to CRE-BSI, characterized by prolonged hospital stays, broad-spectrum antibiotic use, chemotherapy, and immunodeficiency (de Souza et al., 2024).

Our findings demonstrate a markedly higher level of AMR in CRE-BSI compared to that in CSE-BSI. Given the high resistance of CRE-BSI to conventional antibiotics yet their preserved susceptibility to tigecycline and colistin, combination therapy emerges as the optimal strategy and should be tailored to the individual patient’s condition.

Statistically significant factors identified in the multivariate analysis of both the CRE-BSI and CSE-BSI groups represent risk factors for *Enterobacterales*-BSI. In contrast, factors unique to the CRE-BSI analysis constitute specific risk factors for CRE-BSI. Similarly, factors significant only in the CSE-BSI analysis define specific risk factors for CSE-BSI (Kaye et al., 2005). Further, exposure to specific antibiotics (third-generation cephalosporins, carbapenems, and quinolones) and glucocorticoids, along with a history of surgery and gastric tube insertion, emerged as independent risk factors for CRE-BSI. In contrast, surgical history was established as an independent risk factor for *Enterobacterales*-BSI.

A systematic review and meta-analysis by Zhu et al. showed that common risk factors for CRE infection included severe underling diseases, prior antibiotic use (carbapenems, aminoglycosides, quinolones, vancomycin, glycopeptides), ICU admission, invasive procedures (central venous catheter use, mechanical ventilation, tracheostomy, urinary catheter use), and longer LOS (Zhu et al., 2020). We confirmed that antibiotics use, surgery history, and gastric tube insertion increased the risk of CRE infection, while identifying carbapenemase genes (notably blaKPC-2 and blaNDM) as the dominant resistance mechanism among CRE strains in China. Genes encoding carbapenemases are often located on mobile genetic elements, and exposure to carbapenems can promote the emergence of these resistance genes (Han et al., 2020). In contrast to some previous findings, our results demonstrated that third-generation cephalosporin use was a significant risk factor for CRE-BSI, this suggests a broader selective pressure for CRE, indicating that resistance can be promoted by exposure to non-carbapenem antibiotics. Thus, enhancing inpatient antibiotic stewardship and implementing high-dose therapy for a controlled duration is a superior strategy for limiting infection risk (Jeon et al., 2008). Although numerous studies have investigated the risk factors for CRE infection, prior use of quinolones is an independent risk factor remains unclear (Satlin et al., 2017; Stuever et al., 2022; Zuo et al., 2021). Exposure to quinolones promotes bacterial selective pressure, driving resistance not only to this drug class but also to carbapenems via multiple mechanisms. Quinolones can upregulate the expression of the MexEF-OprN efflux pump, triggering a deficiency in OprD—an outer membrane porin—which induces resistance to multiple antibiotics, including carbapenems (Wang et al., 2018). Furthermore, plasmids that mediate quinolone resistance may concurrently carry carbapenemase genes, such as KPC, further contributing to the emergence of carbapenem-resistant strains (Zhu et al., 2020). Unlike previous reports, our study indicated that exposure to glucocorticoid was a significant risk factor for CRE-BSI, possibly due to patients with CRE-BSI requiring glucocorticoid therapy for severe underlying diseases. By disrupting the intestinal microenvironment through eliminating susceptible bacteria and promoting resistant strains, glucocorticoid treatment thereby promotes the evolution of opportunistic microbes into pathogens. Therefore, strengthening antibiotic management measures and regular drug resistance monitoring are crucial for avoiding unnecessary antibiotic exposure. Surgical history was identified as a risk factor for CRE-BSI and *Enterobacterales*-BSI compared to control. Surgery often prolongs LOS, thereby increasing the risk for nosocomial infection. Furthermore, open wounds serve as portals that can elevate the risk of any type of infection (Lavigne et al., 2021), further highlighting the critical role of aseptic techniques in patient care. Gastric tube insertion, another significant risk factor, can provide a suitable place for CRE colonization, and can damage the gastrointestinal mucosa, thereby enhancing the potential for opportunistic bacterial invasion (Tischendorf et al., 2016). Furthermore, gastric tube insertion is often indicated for patients with gastrointestinal dysfunction and compromised nutritional status. This high-risk profile may promote bacterial translocation from the gut, thereby increasing susceptibility to pathogens such as CRE.

CRE are of epidemiological interest for their association with poor outcomes and ability to spread rapidly throughout hospitals (Van Duin et al., 2020). Patients with CRE-BSI demonstrated a significantly higher 30-day mortality than those with other infections. S Sabino et al. reported that the 30-day mortality for CRE-BSI was 63.8% (Sabino et al., 2019), which was higher than that in our study (25.7%). Several factors were independent risk factors for 30-day mortality, including patient group, age ≥ 65 years, respiratory diseases, and digestive system diseases. Several studies have reported that the overall mortality rate in patients with CRE-BSI ranged from 37% to 65%, and some studies have shown that CRE infection was an independent risk factor for mortality (Kedišaletše et al., 2023; Li and Ye, 2017; Sabino et al., 2019). Similarly, patients with CRE-BSI exhibited higher mortality and longer hospital stays in our study, consistent with a previous study (Strich et al., 2020), we observed that the 30-day mortality of CRE-BSI was frequently associated with underlying respiratory diseases in patients. Patients with respiratory diseases are susceptible to both airway colonization and subsequent CRE-BSI (Liu et al., 2012). Patients with CRE-BSI who died within 30 days were significantly older than survivors. Potential reasons for this may include more frequent healthcare exposure and antibiotic use among older adults. Patients with digestive system diseases—who typically exhibit gastrointestinal dysfunction and compromised nutritional status—may require gastric tube insertion. In critically ill patients, this procedure can damage the mucosal barrier, promote bacterial translocation from the gut, and thereby increase the risk of CRE-BSI (Correa et al., 2013; Munoz-Price et al., 2010). Thus, it is imperative to implement stricter infection prevention and control strategies to prevent transmission within hospitals and employ robust antimicrobial stewardship to ensure appropriate antibiotic use, thereby reducing the selection pressure for resistant organisms (Pallares et al., 2022).

Arterial catheterization emerged as a significant risk factor for in-hospital mortality among CRE-BSI patients. This finding reinforces that invasive procedures increase mortality by damaging the mucosal barrier and enabling bloodstream invasion, underscoring the necessity of aseptic technique for infection prevention.

Several studies have established CRE colonization as an independent risk factor for subsequent CRE infection. Therefore, it is essential to strengthen active screening for CRE to facilitate early detection, early isolation, and early intervention in high-risk departments and patients (Chen et al., 2022; Sun et al., 2021). However, the WHO and the US CDC recommend screening for CRE based on local epidemiological conditions (Centers for Disease Control and Prevention, 2015; World Health Organization, 2017). Nosocomial CRE-BSI cases in our hospital were concentrated in the ICU and among patients with specific risk factors, thus active CRE screening should be implemented for these high-risk departments and patient populations.

Clinical outcomes vary considerably among different antimicrobial regimens, and an effective therapeutic approach for CRE-BSI remains elusive. Studies have demonstrated that combination antimicrobial therapy is preferred over monotherapy, particularly in severely-ill patients (Kumar et al., 2010). In our study, no difference was observed between survivors and non-survivors with CRE-BSI treated with monotherapy and combination therapy, consistent with a previous study (Xiao et al., 2018). This is primarily attributable to the complex causes of mortality in patients with CRE-BSI and the lack of an optimal treatment regimen for CRE-BSI. In administering anti-infective therapy, clinicians should consider patients’ individual conditions (including underlying diseases) and their risk factors for mortality, to guide adequate early empirical antibiotic therapy—a critical factor in reducing mortality.

This study is subject to several limitations. Its single-center, retrospective design may affect the generalizability of the findings and could introduce potential information and selection biases. Furthermore, the absence of carbapenemase gene detection means some carbapenem-susceptible carbapenemase-producing isolates may have been misclassified as CSE. Future studies employing molecular analyses will help draw more precise conclusions.

## 5 Conclusion

This matched case-case-control study delineated distinct risk factor profiles for bloodstream infections. Surgical history was established as an independent risk factor for *Enterobacterales*-BSI, while exposure to specific antibiotic classes (third-generation cephalosporins, carbapenems, and quinolones) and glucocorticoids, along with surgical history and gastric tube insertion, were independently associated with CRE-BSI. Furthermore, several factors predicted increased mortality, including CRE-BSI itself, respiratory diseases, age ≥ 65 years, digestive system diseases and arterial catheter are more likely to have higher mortality. Notably, no significant differences in outcomes were observed across various antimicrobial regimens. Consequently, early identification of these risk factors is crucial, and primary prevention strategies—including antimicrobial stewardship, reduced hospitalization, and minimized invasive interventions—are essential to reducing the incidence and mortality of CRE-BSI. These findings provide critical insights to guide effective antimicrobial therapy development and prevention of CRE-BSI.

## Data Availability

The original contributions presented in the study are included in the article/supplementary material. Further inquiries can be directed to the corresponding authors.
